# Osborne Fractionation of Steam-Distilled Residue of Coriander (*Coriandrum sativum* L.) Seeds: Going Beyond Proteins

**DOI:** 10.3390/foods15213993

**Published:** 2026-11-07

**Authors:** Petya Ivanova, Radka Vrancheva, Anton Slavov, Vesela Chalova

**Affiliations:** 1 Department of Biochemistry and Nutrition, University of Food Technologies, 26 Maritsa Blvd., 4002 Plovdiv, Bulgaria; p_georgieva@uft-plovdiv.bg; 2 Department of Analytical Chemistry and Physical Chemistry, University of Food Technologies, 26 Maritsa Blvd., 4002 Plovdiv, Bulgaria; r_vrancheva@uft-plovdiv.bg; 3 Department of Organic Chemistry and Inorganic Chemistry, University of Food Technologies, 26 Maritsa Blvd., 4002 Plovdiv, Bulgaria; a_slavov@uft-plovdiv.bg

**Keywords:** coriander seeds distilled residue, proteins, Osborne fractionation, antioxidant properties

## Abstract

Steam-distilled residue of the coriander seeds (SRC) is a by-product remaining after steam distillation of essential oils from seeds of *Coriandrum sativum* L. Although rich in valuable compounds, the non-volatile lipid and protein fractional profiles in this biomaterial are insufficiently investigated. The aim of the study was to evaluate the extractability of protein in SRC and defatted SRC (DSRC) by the Osborne fractionation approach, evaluate total phenolic and flavonoid contents, and the antioxidant activity of the protein fractions by five different methods: DPPH, FRAP, ABTS, CUPRAC, and hydroxyl radical scavenging assay. The co-produced non-volatile fatty fraction, obtained by n-hexane extraction from SRC, was qualitatively and quantitatively characterized. The results indicated that the treatment of SRC with n-hexane not only generated a potentially functional product, but also improved the subsequent protein extraction. All protein fractions, water-soluble albumins, salt-soluble globulins, alcohol-soluble prolamins, and alkaline-soluble glutelins obtained from the DSRC, contained higher amounts of protein, as the total protein yield reached 22.62% compared to a 16.67% total protein yield obtained from the SRC. All protein fractions exhibited antioxidant properties as evaluated by the five methods, with the albumin fraction being superior. In all other evaluations, except for the albumin fraction, examined by the hydroxyl radical scavenging assay, the protein fractions obtained from the DSRC exhibited higher antioxidant potency. The lipid-containing derivative, obtained by n-hexane extraction of SRC, prevailed in monounsaturated (76.12%) and polyunsaturated fatty acids (15.22%), with petroselinic acid being the main component of the lipid mixture (67.31%). The presented data supported improved protein extractability following defatting, the presence of phenolic and flavonoid compounds in the Osborne extracts, and the in vitro antioxidant activity of the latter. The study demonstrated the potential of SRC upcycling to multiple value-added derivatives as an alternative for more efficient and comprehensive utilization of this still undervalued biomaterial.

## 1. Introduction

Coriander (*Coriandrum sativum* L.) is an annual plant widely cultivated throughout the world, with India being the main producer and exporter [1]. Due to its chemical composition and flavor properties, almost all parts of the plant, leaves, stems, and seeds, are usable with diverse applications in the food, pharmaceutical, chemical, perfumery, and cosmetics industries [2]. Among them, coriander seeds are mainly valued for their oil content and composition, which may vary depending on various factors including variety, stage of seed maturity and harvest time, climate conditions, soil fertility, and agronomic practices [3,4,5]. The total amount of lipids may reach up to 17–20%, with 1.8% of them being volatile oil. In addition, coriander seeds are rich in proteins (11–13%), starch (11%), and crude fiber (30%) [6]. They also contain phenolics (246.14 mg GAE/100 g), flavonoids (94.27 mg QE/100 g), and minerals such as Mn, Mg, Fe, Zn, and Ca [7].

While essential oils can be obtained by using different solvents and modern extraction approaches, steam distillation is still considered an industrial standard for the production of high-quality essential oil from coriander seeds. The process results in the generation of high-purity essential oil containing 60–70% linalool as a primary product and a residual biomass as a by-product, while still being environmentally friendly [8]. The removal of the oil from the seeds enhances the contents of non-volatile components like crude fiber, minerals, bioactive compounds, triglycerides, and proteins in the by-product, thus turning the biomaterial into a valuable source for either direct use in feed [9,10,11] and food [12] or preparation of functional derivatives for the food industry [13]. Although both directions have been explored to some extent, little is known about proteins and non-volatile lipids remaining in the steam-distilled residue of the coriander seeds (SRC) and their potential application. Previous studies demonstrated that the coriander press cake lipid fraction was rich in monounsaturated fatty acids (MUFAs) and polyunsaturated fatty acids (PUFAs), with petroselinic acid (C18:1n-6), a potent anti-inflammatory and antimicrobial agent, being present in the highest amount in the MUFA composite (75.5–77.2%) [14,15]. With the constant increase of the worldwide human population, although difficult, studies on efficient utilization of by-products of the oil-producing industry, especially those providing nutritive ingredients such as proteins and fats, are needed.

Furthermore, the Osborne fractionation approach allows for the extractability evaluation and preparation of four fractions of proteins, namely water-soluble albumins, salt-soluble globulins, alcohol-soluble prolamins, and alkaline-soluble glutelins. It is a sequential extraction technique that separates plant proteins based on their solubility in different solvents, namely water, 5% NaCl, 70% ethanol, and 0.1 N NaOH [16]. However, the solvents do not selectively extract only proteins. Non-proteinaceous compounds such as phenolics, flavonoids, and microelements might also be co-extracted with the proteins, thus attributing potential bioactive properties to the protein fractions.

The aim of the current study was to evaluate the extractability of protein in SRC and defatted SRC (DSRC) in four sequential extraction steps, generating albumins, globulins, prolamins, and glutelins fractions. The fractions were characterized in terms of total content of phenolic compounds, flavonoids, and antioxidant capacity evaluated by five different methods, namely 2,2-diphenyl-1-picrylhydrazyl radical scavenging (DPPH), Ferric Reducing Antioxidant Power (FRAP), 2,2′-azino-bis (3-ethylbenzothiazoline-6-sulfonic acid (ABTS), Cupric Ion Reducing Antioxidant Capacity (CUPRAC), and hydroxyl radical scavenging activity. Although proteins in SRC and DSRC were the main focus of investigation in this study, non-volatile fatty acids extracted from the SRC as a co-product were also quantitatively and qualitatively characterized to provide insight into their potential application as a SRC derivative in the food industry or/and cosmetics.

## 2. Materials and Methods

### 2.1. Material

**Figure 1:**
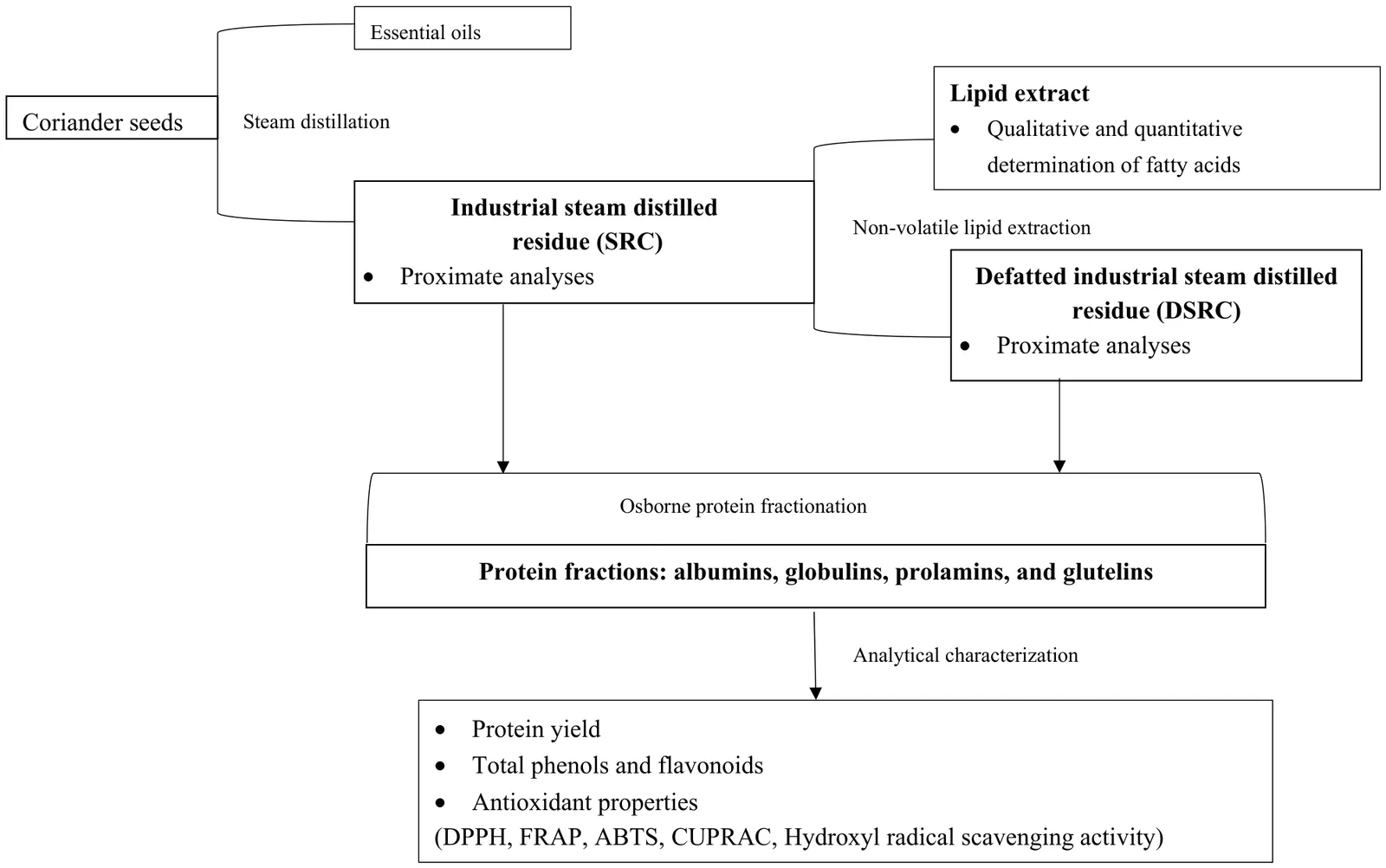
Workflow of experimental design.

Steam-distilled residue of coriander (*Coriandrum sativum* L.) seeds (SRC) was provided by “Berje Trakia” Ltd. (Starosel, Hisarya, Bulgaria). The biomaterial remained as a by-product after industrial extraction of essential oils by steam distillation of ripened and partially dried fruits of the *C. sativum* herb, produced in 2025. The process was conducted at 100 °C for 2 h. The SRC was further defatted under laboratory conditions by using n-hexane as a solvent. The extraction of the crude lipid fraction was carried out twice at room temperature with a hydro-module of 1:10 (100 g and 2 × 1000 mL n-hexane). The resulting solid coriander residue was spread into a thin layer and dried at room temperature (25 °C) to obtain defatted steam-distilled residue of coriander seeds (DSRC). The n-hexane extract was evaporated on a rotary vacuum evaporator at 40 °C (RV 3 V Rotary Evaporator, IKA@Werke GmbH & Co. KG, Staufen, Germany) to remove the n-hexane and obtain the lipid fraction. The lipid fraction, SRC, and DSRC were collected and stored in closed containers in a fridge (4–5 °C) until analysis. The workflow of the experimental design is presented in Figure 1. All reagents used were of analytical grade and purchased from FILLAB Ltd., Plovdiv, Bulgaria.

### 2.2. Chemical Analyses

Total nitrogen was determined by the Kjeldahl method and multiplied by 6.25 to convert to crude protein [17]. The amounts of total lipids and carbohydrates were evaluated as described by Bligh and Dyer [18] and Dubois et al. [19], respectively. Ash content was determined by ICC Standard №104/1 [20].

### 2.3. Quantitative and Qualitative Determination of Fatty Acids in Lipid Fraction

Gas Chromatography-Flame Ionization Detection (GC-FID) was used to evaluate the fatty acid composition of the lipid fraction obtained from SRC. The analysis was performed on a gas chromatograph (model TRACE 1610 GC, Thermo Fisher Scientific, Rodano, Italy) with an AI 1310 series autosampler and a TR-FAME column (Thermo Fisher Scientific, Rodano, Italy): 100 m × 0.25 mm, 0.20 µm film thickness. Nitrogen was used as a carrier gas with a constant flow of 1 mL/min. Injector temperature was 240 °C, while the FID detection temperature was set at 260 °C. The temperature parameters used were as follows: target value 100 °C, hold time 0.2 min; and from 100 °C to 240 °C at a heating rate of 2 °C/min and holding at 240 °C for 25.00 min. The amounts of detected components were determined using Chromeleon 7 software (Thermo Fisher Scientific, Rodano, Italy) based on the area of the peaks from the GC/FID chromatogram and internal normalization. The identification of the fatty acids was carried out based on the retention times of corresponding standards [21]. Lipid quality indices, atherogenicity index (AI), thrombogenicity index (TI), hypocholesterolemic to hypercholesterolemic fatty acids ratio (H/H ratio), calculated oxidizability (Cox), and oxidative susceptibility (Os) were calculated by following Chen et al. [22] and M’Rabet et al. [23].

### 2.4. Protein Fractionation

SRC and DSRC were sequentially extracted with water, 5% NaCl, 70% ethanol, and 0.1 N NaOH to obtain albumin, globulin, prolamin, and glutelin fractions, respectively. Each fractional extraction was repeated three times with a decreasing volume of solvent (mL) from the first to the third extraction as follows: 1:10, 1:5, and 1:2.5 weight-to-volume ratios [16]. All extractions were performed at room temperature (23 °C) for 30 min under constant agitation. Extraction aliquots were collected and protein contents were evaluated by the Bradford method [24]. The respective solvents, instead of the extracts, were used as controls. Bovine serum albumin was used for the generation of a standard curve. Protein yield was calculated from the total mass of protein recovered in each extract relative to the initial protein mass, accounting for the different extraction volumes. More precisely, the protein yield (Y%) was presented as a fraction of the amount of the crude protein in a sample determined by the Kjeldahl method [17] using the following equation:

(1) $$Y=\frac{A}{B}\times 100$$

where *A* (%) is the protein content in the extract, and *B* (%) is the protein content in SRC or DSRC. The ratio was multiplied by 100 to express the result as a percentage.

### 2.5. Determination of Phenolic and Flavonoid Contents in SRC and DSRC Protein Fractions

The total phenol content in SRC and DSRC protein fractions was evaluated using Folin–Ciocalteu reagent [25] as described by Ivanov [26]. Calculations were made using a standard curve prepared with gallic acid. Total flavonoids were estimated following the procedure of Kivrak et al. [27], as quercetin was used for the preparation of a standard curve. Contents were calculated on a dry matter basis: 92.33 ± 0.11% for SRC, and 92.24 ± 0.03% for DSRC. The dry matter was determined with a moisture analyzer KERN DAB 100-3 (Kern & Sohn GmbH, Balingen, Germany).

### 2.6. Determination of Antioxidant Activity

#### 2.6.1. Scavenging Effect on 2,2-Diphenyl-1-picryl hydrazyl (DPPH) Radical

The antioxidant potential of SRC and DSRC protein fractions against DPPH was evaluated as described by Dimov et al. [28] with some modifications. Briefly, 0.15 mL of each extract was mixed with 2.85 mL 0.06 mM DPPH fresh solution in 96% ethanol. The mixtures were kept at room temperature in darkness, and the absorbance (A) was measured at 517 nm with a spectrophotometer (Spekol 11; Carl Zeiss Jena, Jena, Germany) after 30 min. Antioxidant radical scavenging activity (AA) was calculated using the formula AA = (Acontrol − Asample)/Acontrol and multiplied by 100 to express it in percentage. The control was treated as the corresponding sample but using the same amount of the respective solvent instead.

#### 2.6.2. Ferric Reducing Antioxidant Power (FRAP) Assay

FRAP was performed as described by Ivanov et al. [26]. Aliquots of three ml freshly prepared FRAP reagent (10 parts 0.3 M acetate buffer (pH 3.6), 1 part 10 mM 2,4,6- tripyridyl-s-triazine (TPTZ) in 40 mM HCl, and 1 part 20 mM FeCl_3_·6H_2_O in deionized H_2_O) were mixed with 0.1 mL of SRC or DSRC extracts. The reaction time was 10 min at 37 °C in darkness, followed by measurement of the absorbance at 593 nm against a blank prepared with the corresponding sample solvent. A standard curve was built with FeSO_4_·7H_2_O. The results of FRAP analysis were expressed as µmol Fe^2+^ equivalents per gram dry weight SRC or DSRC [29].

#### 2.6.3. Hydroxyl Radical Scavenging Activity

Hydroxyl radical scavenging capacity of SRC and DSRC protein fractions was evaluated as described by Chalova et al. [30]. Briefly, the reaction mixture contained 100 μL of 28 mM 2-deoxy-D-ribose (dissolved in 10 mM KH_2_PO_4_–K_2_HPO_4_ buffer, pH 7.4), a 500 μL sample protein fraction, 200 μL of 200 μM FeCl_3_ and 1.04 mM ethylenediaminetetraacetic acid mixed (1:1 *v*/*v*) before addition, 100 μL of 10 mM H_2_O_2_ and 100 μL of 1 mM ascorbate freshly prepared with de-aerated water. After incubation for 1 h at 37 °C, 1 mL thiobarbituric acid (1% in 50 mM NaOH) and 1 mL trichloroacetic acid (2.8% in water) were added, and the tubes were boiled for 20 min. The absorbance (A) of the developed pink chromogen was measured at 532 nm after cooling against an appropriate blank (containing only buffer and deoxyribose). The inhibition (%) was calculated by the formula I (%) = 100 (Asample/Anegative control) × 100. In the negative control, the reaction mixture contained 500 μL of the respective solvent instead. Mannitol was used as a positive control [31].

#### 2.6.4. ABTS (2,2′-Azino-bis(3-ethylbenzothiazoline-6-sulfonic acid) Assay

The ABTS method described by Thaipong et al. [32] and Vrancheva et al. [33] was used. ABTS radicals were generated by mixing aliquot parts of 7.0 mM 2, 2′azinobis (3)- ethylbenzthiazoline-6-sulfonic acid (ABTS) in distilled water and 2.45 mM potassium persulfate (Merck, Darmstadt, Germany) in deionized water. The reaction was performed for 16 h at ambient temperature in darkness and the generated ABTS radical was stable for several days. Before analysis, 2.0 mL of the generated ABTS^•+^ solution was diluted with methanol at a 1:30 (*v*/*v*), so the obtained final absorbance of the working solution was about 1.0 to 1.1 at 734 nm. For the assay, 2.85 mL of this ABTS ^•+^ solution was mixed with 0.15 mL of the obtained protein extractions. After 15 min at 37 °C in darkness, the absorbance was measured at 734 nm against the corresponding solvent. The antioxidant activity was expressed as mM Trolox equivalents (TE)/g DW using a calibration curve, built in the range from 0.05 to 0.5 mM Trolox dissolved in methanol.

#### 2.6.5. Cupric Reducing Antioxidant Capacity (CUPRAC) Assay

The assay was performed according to Apak et al. [34] and Vrancheva et al. [33]. The reaction was started by mixing 1.0 mL 10 mM CuCl_2_.2H_2_O in distilled water, 1.0 mL 7.5 mM neocuproine in methanol, 1.0 mL 0.1 M ammonium acetate buffer (pH 7.0), 0.1 mL of the investigated protein fraction, and 1.0 mL of deionized water. A blank sample, with the corresponding solvent instead of the protein fraction, was prepared as well. The reaction was carried out for 20 min at 50 °C in darkness and the sample absorption (450 nm) was recorded against the blank. Antioxidant activity was expressed as mM (TE)/g DW using a calibration curve, generated in the range from 0.05 to 0.5 mM Trolox dissolved in methanol.

### 2.7. Statistical Analyses

Analyses were performed in triplicates representing three independent fractionation experiments. Results are presented as means ± standard deviation (SD). Data were analyzed by one-way analysis of variance (ANOVA) using the Statgraphics Centurion statistical program (version XVI, 2009) (Stat Point Technologies, Inc., Warrenton, VA, USA). Mean differences were established by Fisher’s least significant difference test for paired comparison with a significance level α = 0.05.

## 3. Results and Discussion

### 3.1. SRC and DSRC Composition

Spice by-products, although often discarded as waste, are rich in valuable compounds which, after extensive characterization, might be taken into new pathways of utilization [35]. The SRC, remaining after the removal of the essential oils from coriander seeds, contained substantial amounts of macro-components (Table 1). The total lipids comprised 13.11% of SRC, which were reduced to 7.97% in the DSRC after extraction with n-hexane. The process increased the amounts of the remaining macro-components in DSRC, as the crude protein and total carbohydrate contents increased from 15.23% to 17.89% and 35.23% to 37.17%, respectively. By following the classical approach with n-hexane treatment, 39.20% of the non-volatile lipid fraction in the SRC was extracted (Table 1). The main idea behind the study was to check the possibility for utilization of SRC and DSRC as a source of proteins. At the same time, defatting the SRC by extraction with n-hexane served for examination of the lipids in the SRC. Further work and considerations will be taken into account when applications are examined.

With the relatively high protein contents, both SRC (15.23%) and DSRC (17.89%) could be considered a good source of protein. Our data agreed with the results reported by Aleshin et al. [10], who evaluated the potential of coriander cake as a feed ingredient. By studying four coriander cakes, provided by two different oil manufacturers, the authors established variability in the crude protein content from 14.18 to 16.01%, which was attributed to climate and regional features of the seed origin. It was also speculated that the coriander cakes, with essential amino acids reaching 4.83%, might have a positive influence on cow metabolism and reproductivity. The inclusion of 10 to 20% coriander cake in the diets of lactating goats reduced the level of easily digestible carbohydrates needed, shifting the protein source inclusion in the diets towards an enhanced proportion of non-digestible protein yet increasing the absorption of feed nitrogen that is important for maintaining the physiological needs of the animals and milk productivity [9]. The observed protein recovery in our study is most probably due to unfavorable processing parameters applied to the coriander seeds, such as elevated temperature or/and use of organic solvents that denature proteins and favor their interaction with the other compounds in the matrix. Furthermore, as this is a first study on Osborne protein fractionation from SRC and DSRC, full optimization of the extraction parameters and protein recovery model has not been performed, as it requires a dedicated separate study with a purposive experimental design. Most cereals, rice, wheat, corn, barley, and pseudo-cereals (quinoa, amaranth, buckwheat) do not contain more than 15% crude protein but are considered superior for human diet formulation than the meal/cakes of the oil-producing industry because of their better digestibility [36].

As both SRC and DSRC contained high amounts of total carbohydrates (35.23%, 37.17%, Table 1), conferring potential application, this group of macro-components deserves detailed attention and will be investigated in a separate study.

### 3.2. Fatty Acid Profile of SRC Non-Volatile Lipid Fraction

The fatty acid profile of the n-hexane-extracted non-volatile lipid fraction of SRC demonstrated prevailing monounsaturated fatty acids (76.12%, MUFA) followed by polyunsaturated fatty acids (15.22%, PUFA) and saturated fatty acids (4.37%, SFA) (Table 2). Our findings agree with previously reported trends in the fatty acid composition of coriander seeds or cakes [6,14,15]. Similarly, petroselinic acid (C18:1 n-6) in the non-volatile lipid fraction of the SRC was present in the highest amount (67.31%) in MUFA, followed by oleic acid (C18:1 n-9, 8.61%). The chromatogram of the coriander fatty acid profile (Supplementary Materials) showed that two peaks appeared around 57.01 and 57.122 min, which is typical for methyl esters of C18:1. The column used was 100 m, which allowed the separation of the two isomers. The second one (at 57.122 min, the smaller one) corresponds to methyl oleate. The first one (at 57.01 min, the larger one) was tentatively assigned as methyl petroselinate based on the literature data [37] and on the assumption that fatty acid methyl esters with a double bond closer to the ester group are leaving the column earlier (oleic acid—C18:1 n-9; petroselinic acid—C18:1 n-6). Furthermore, the standard of methyl petroselinate (compared with methyl oleate) confirmed its presence in the coriander lipids.

In general, petroselinic acid is abundant in plants of the Apiaceae family such as dill (*Anethum graveolens*), celery (*Apium graveolens*), caraway (*Carum carvi*), cumin (*Cuminum cyminum*), and fennel (*Foeniculum vulgare*) [38]. It is an isomer of oleic acid (C18:1, n-9), which attributes better oxidative stability to petroselinic acid-rich oil [23]. The same authors reported a wide use of petroselinic acid in cosmetics and dermatology as a constituent of lotions and creams, in pharmaceuticals as an antimicrobial agent, in the food industry as a natural emulsifier in sauces and dressings, and in the chemical industry via the products of its oxidative cleavage, adipic acid and lauric acid. Therefore, the by-product SRC could be potentially upcycled to the production of petroselinic acid as a value-added compound enabling more efficient and comprehensive utilization.

Regarding the quality of the extracted non-volatile lipid fraction for SRC, data in Table 2 show a relatively low amount of SFA (4.37%) and, as a consequence, a high PUFA-to-SFA ratio (3.48).

The ratio is higher than 0.45, as recommended by Briggs et al. [39], indicating potential health benefits from the inclusion of the SRC or the non-volatile lipid fraction in the human diet. The relatively high amounts of MUFA and PUFA and the low level of SFA in the SRC lipid fraction explained the low values of AI (0.07) and TI (0.09) parameters (Table 2), which imply a potential for reduction in the total cholesterol level and clot formations in blood vessels. In addition, a good indicator of the SRC fat profile is the high H/H ratio (21.34, Table 2), which is considered nutritionally beneficial as it indicates the prevalence of unsaturated, hypocholesterolemic fatty acids, compared to saturated, hypercholesterolemic fatty acids. The Cox (2.34) and Os (770.37) indices imply relatively high oxidative stability, which, however, should be further proven by direct oxidation measurements. These indices indicated lower susceptibility to oxidative deterioration compared to other edible species from Apiaceae, as evidenced by their Cox (2.91–9.75) and Os (1072–4444) values [38]. Although data on the non-volatile lipid fraction of SRC imply potential health benefits, they are not direct evidence of physiological or clinical effects of the recovered lipid fraction.

### 3.3. Protein Extractability

By using sequential extraction of proteins from SRC and DSRC with water, 5% NaCl, 70% ethanol, and 0.1 N NaOH, four different fractions were obtained, namely albumins, globulins, prolamins, and glutelins. Decreasing the amount of solvent during the three sequential extractions in the Osborne protein fractionation was chosen to achieve an enhanced concentration of extracted compounds per milliliter of liquid, which facilitates further analyses with the extracts. Although the Kjeldahl method is a standard method for protein quantification in dry samples, it is time-consuming, labor-intensive, and expensive. Being significantly faster and simpler, the Bradford method was employed as an alternative for high-throughput protein evaluation in the numerous extracts generated under laboratory conditions, as it was in our study.

The lowest protein yield was obtained for prolamins (1.59% for SRC and 2.42% for DSRC), while the highest ones were achieved when using 0.1 N NaOH as a solvent, with a 7.47% protein yield in SRC and 9.84% in DSRC (Table 3). Higher protein yields for all protein fractions were observed in DSRC compared to SRC (Table 3). The total protein yield from DSRC reached 22.62%, while that from the SRC (16.67%) was significantly lower (*p* < 0.05) (Table 3).

In fact, the extraction of the non-volatile lipid fraction from SRC resulted not only in the generation of a product with added value (non-volatile lipid fraction), but also in an improvement of protein extractability. According to Waszkowiak et al. [40], in cells, lipids tend to interact with proteins to form lipoprotein complexes, which are often less soluble and harder to extract. In oil-bearing seed-processed by-products, lipids can physically coat protein bodies, acting as a hydrophobic barrier that prevents extraction solvents from reaching the protein. This might also explain the higher glutelin yield, as the solvent used for their extraction, NaOH, solubilizes lipids, thus liberating a higher amount of extractable protein [41].

For all protein fractions, the first extraction yielded the highest amounts of protein. The second extraction yielded approximately 50% of the protein extracted by the first one, indicating the necessity of repeating the extraction procedure. While the samples were not completely protein-exhausted after the second extraction, the yields obtained from the third extraction were relatively low (Table 3). In contrast, the majority of protein yield from rapeseed seeds, rapeseed meal, and ethanol-treated rapeseed meal was achieved with the first extraction [16]. The results indicate a potential omission of the third extraction from the overall process, if the reduction in the total protein yield is compensated by a decrease in the expenses associated with the extraction step. However, as no process-efficiency or techno-economic assessment was performed, a possible future process-optimization strategy is needed for a confirmatory conclusion.

Proteins in Osborne-derived fractions have different nutritive profiles and functional properties, which makes their potential application versatile and valuable. As the protein fractional profile of both SRC and DSRC has not been studied, the findings of current research would be useful in strategies related to their potential use as plant protein sources.

### 3.4. Total Phenolic and Flavonoid Contents in SRC and DSRC Protein Fractions

Phenolic compounds and flavonoids are plant secondary metabolites with diverse structures and biological activities, including antioxidant potency [42]. Recently, they have gained popularity due to their health-promoting benefits, as shown by Cosme et al. [43] and versatile applications in industrial sectors [44]. In plants, polyphenols might interact with proteins via covalent or non-covalent bonds to form complexes with higher molecular weight [45]. According to the same authors, the solubilization of the complex is influenced by the concentration of polyphenols, their molecular weight and structure, pretreatment of the biomaterial, and type of the solvent.

In both SRC and DSRC, the highest phenolic amounts were co-extracted with albumins, followed by the glutelin fraction (Table 4). Reis et al. [46], who studied phenolics and flavonoids extractability from apple pomace, established that chlorogenic acid, feruloylquinic acid, epicatechin, and procyanidins were extracted in considerable amounts when using water as a solvent.

The high content of phenolics in the glutelin fraction was not a surprise. Extracting phenolics with sodium hydroxide is possible due to a reversible acid–base reaction [47]. The aqueous sodium hydroxide deprotonates the weak phenolic acids, creating water-soluble particles that are separable from non-acidic organic compounds in the mixture.

Lower phenolic contents were established in prolamin fractions obtained by extraction with 70% ethanol (Table 4), although ethanol is frequently used for the recovery of polyphenols [48]. However, the same authors reported that the extraction efficiency is structure-dependent and is not the same for the different phenolic compounds. For example, some phenolics were found to be more soluble in polar solvents like alcohols (ethanol and methanol), but other compounds, such as gallic acid, are more soluble in water.

Although some significant statistical differences in the phenolic contents of the corresponding protein fractions of SRC and DSRC were found, they were low when compared by absolute values (Table 4). The Folin–Ciocalteu assay is broadly used for the quantification of phenolic compounds, but it should be noted that the reagent is not entirely specific for them and can respond to other reducing substances that may be present in complex protein-containing extracts.

Similar trends were observed with the flavonoid contents in protein fractions of SRC and DSRC (Table 5).

### 3.5. Antioxidant Capacity of SRC and DSRC Protein Fractions

Phenolics and flavonoids are known as potent antioxidants, as this feature is related to their structural integrity [42]. Therefore, the processing of biomaterials might be expected to alter the biological activity of these compounds. This is especially valid for biomaterials being steam-exposed, such as the SRC used in the current study. While mild treatment of the biomass might be beneficial for phenolics recovery [49], prolonged or high-heat steam exposure can destroy or polymerize phenolics, reducing their total levels and antioxidant benefits [42]. As no similar study has been performed so far, five methods differing in their principles, namely DPPH, FRAP, hydroxyl radical scavenging properties, ABTS, and CUPRAC, were used to obtain as much as possible detailed information about the antioxidant properties of the protein fractions obtained from SRC and DSRC.

The albumin fraction exhibited the highest antioxidant activity by all methods used (Table 6, Table 7, Table 8, Table 9 and Table 10).

This might be related to the highest contents of phenolics and flavonoids established in this fraction (Table 4 and Table 5). Decreasing antioxidant activity with the reciprocal number of extractions could also be linked to the levels of the phenolics and flavonoids in these extracts (Table 4 and Table 5). Except for DPPH evaluation (Table 6), the glutelin fraction exhibited the second-highest antioxidant capacity when measured by FRAP (Table 7) and CUPRAC assays (Table 10), which corresponded to its higher total phenolic and flavonoid contents (Table 4 and Table 5). The antioxidant activity of the glutelin fraction was not evaluated by the hydroxyl radical scavenging and ABTS methods (Table 8 and Table 9) due to interference with the high-concentration NaOH solution (0.1 N) used for the preparation of these extracts. The prolamin fraction was also not evaluated by the hydroxyl radical scavenging assay, as ethanol is a hydroxyl radical scavenger by itself and completely bleached the experimental reaction.

When DPPH was applied, higher antioxidant activity was demonstrated by the prolamin fraction compared to the glutelin one (Table 6), although the total phenolics and flavonoid contents were higher in the latter (Table 4 and Table 5). It is difficult to speculate on the reason for this finding, since there is no information on phenolic and flavonoid compositions in the SRC and DSRC. Phenolics and flavonoids with structures favoring the DPPH reaction may be in higher amounts in the prolamin fractions than in the glutelin ones.

Except for the albumin fraction examined by the hydroxyl radical scavenging assay (Table 8), in all other evaluations, the protein fractions obtained from the DSRC exhibited higher antioxidant properties (Table 6, Table 7, Table 9 and Table 10). While no relation to the phenolic and flavonoid contents could be made in this case, the higher levels of proteins and ash in the DSRC might contribute to the overall antioxidant activities of the protein fractions. The side chains of the amino acids tyrosine, cysteine, tryptophan, and histidine presented in the peptide/polypeptide chain exhibit antioxidant features by either hydrogen atom transfer (HAT) and/or single electron transfer (SET) mechanisms [50]. Metals such as iron and copper [51], manganese [52], and zinc [53] have an important role in the antioxidant defense mechanism. The findings of Wanasundara et al. [54] also indicated that the amount of phenolic compounds was not the only factor when considering the antioxidant activity. However, the specific contribution of the proteins and metals to the total antioxidant capacities of the SRC and DSRC Osborne extracts has not been experimentally investigated yet, and deserves to be an objective of a separate detailed evaluation.

## 4. Conclusions

SRC, a by-product remaining after steam distillation of essential oils from coriander seeds, was investigated as a source of value-added derivatives. The study demonstrated the potential of the SRC for the generation of multiple products with a possible application in the food industry. Following the experimental workflow, a non-volatile lipid fraction was generated in addition to sequentially prepared protein fractions by the Osborne approach. The lipid-containing derivative was dominated by monounsaturated and polyunsaturated fatty acids, which are considered beneficial for human health. Petroselinic acid, a potent anti-inflammatory and antimicrobial agent, was the main component of the lipid mixture. The n-hexane extraction of lipids from SRC not only resulted in a potentially functional product, but also improved the subsequent protein extraction to obtain albumin, globulin, prolamin, and glutelin fractions with higher amounts of proteins compared to those obtained from SRC. All protein fractions, in addition to being a protein source, demonstrated antioxidant properties as evaluated by DPPH, FRAP, Hydroxyl radical scavenging properties, ABTS, and CUPRAC methods, with the albumin fraction being superior. Furthermore, except for the albumin examined by the hydroxyl radical scavenging assay, in all other evaluations, the protein fractions obtained from the DSRC exhibited higher antioxidant properties. Overall, the present data support improved protein extractability following defatting, the presence of phenolic and flavonoid compounds in the Osborne extracts, and in vitro antioxidant activity. Their nutritional functionality, health effects, and suitability as natural food additives, although plausible, require further experimental evaluation and validation. As the SRC originates from a single industrial source, possible variability associated with cultivar, geographical origin, growing conditions, harvest, and steam-distillation conditions could be assumed in future work.

## Figures and Tables

**Table 1:** Proximate analyses of steam-distilled residue of coriander (SRC) and defatted steam-distilled residue of coriander (DSRC).

Component	Content, %	
	SRC	DSRC
Crude protein	15.23 ± 0.15 ^b^	17.89 ± 0.08 ^a^
Total lipids	13.11 ± 0.87 ^a^	7.97 ± 0.38 ^b^
Total carbohydrates	35.23 ± 0.88 ^a^	37.17 ± 2.16 ^a^
Ash	5.99 ± 0.03 ^b^	7.16 ± 0.14 ^a^

^a,b^ Means in a row with different superscripts differ significantly (*p* < 0.05).

**Table 2:** Fatty acid composition of non-volatile lipid fraction extracted from steam-distilled residue of coriander seeds (SRC).

Fatty Acid Profile	Retention Time, min	Content, %
**Saturated Fatty Acids (SFAs)**		
Palmitic acid, C16:0	48.60	3.42 ± 0.11
Stearic acid, C18:0	55.61	0.85 ± 0.06
Arachidic acid, C20:0	62.10	0.10 ± 0.02
Myristic acid, C14:0	41.06	<0.01
Behenic acid, C22:0	68.10	<0.01
Lignoceric acid, C24:0	73.80	<0.01
**Total SFA**		4.37 ± 0.32
**Monounsaturated Fatty Acids (MUFAs)**		
Petroselinic acid, C18:1 n-6	56.80	67.31 ± 1.60
Palmitoleic acid, C16:1 n-7	50.25	0.20 ± 0.02
Oleic acid, C18:1 n-9	56.96	8.61 ± 0.26
Erucic acid, C22:1 n-9	69.51	<0.01
**Total MUFA**		76.12 ± 1.63
**Polyunsaturated Fatty Acids (PUFAs)**		
Linoleic acid, C18:2 n-6	59.26	15.05 ± 0.63
α-Linolenic acid, C18:3 n-3	61.88	0.17 ± 0.03
**Total PUFA**		15.22 ± 0.73
**Total Fatty Acids Identified**		95.71 ± 1.63
**Fatty Acids Nutritional Indices**		
Atherogenicity index (AI)		0.07
Thrombogenicity index (TI)		0.09
Hypocholesterolemic to Hypercholesterolemic fatty acids ratio (H/H ratio)		21.34
Calculated oxidizability (Cox)		2.34
Oxidative susceptibility (Os)		770.37

**Table 3:** Protein extractability from steam-distilled residue of coriander (SRC) and defatted steam-distilled residue of coriander seeds (DSRC).

Fractions	Extraction №	Protein Yield (Y, %)	
		SRC	DSRC
Albumin	1	2.74 ± 0.06 ^a,B^	3.55 ± 0.10 ^a,A^
	2	1.47 ± 0.02 ^b,B^	2.00 ± 0.02 ^b,A^
	3	0.69 ± 0.02 ^c,B^	0.91 ± 0.0 ^c,A^
	Total	4.90	6.46
Globulin	1	1.67 ± 0.01 ^a,B^	2.32 ± 0.07 ^a,A^
	2	0.71 ± 0.01 ^b,B^	1.03 ± 0.02 ^b,A^
	3	0.33 ± 0.01 ^c,B^	0.55 ± 0.01 ^c,A^
	Total	2.71	3.90
Prolamin	1	0.96 ± 0.00 ^a,B^	1.44 ± 0.04 ^a,A^
	2	0.42 ± 0.01 ^b,B^	0.67 ± 0.00 ^b,A^
	3	0.21 ± 0.00 ^c,B^	0.31 ± 0.01 ^c,A^
	Total	1.59	2.42
Glutelin	1	4.44 ± 0.12 ^a,B^	5.82 ± 0.08 ^a,A^
	2	2.05 ± 0.04 ^b,B^	2.74 ± 0.04 ^b,A^
	3	0.98 ± 0.00 ^c,A^	1.28 ± 0.00 ^c,B^
	Total	7.47	9.84
Total		16.67	22.62

^a–c^ Means in a column for a specific fraction with different superscripts differ significantly (*p* < 0.05). ^A,B^ Means in a row with different superscripts differ significantly (*p* < 0.05).

**Table 4:** Total phenol content (mg GAE/g dry weight) in steam-distilled residue of coriander (SRC) and defatted steam-distilled residue of coriander seeds (DSRC) protein fractions.

Fractions	Extraction №	Content, mg GAE/g Dry Weight	
		SRC	DSRC
Albumin	1	2.62 ± 0.00 ^a,A^	2.14 ± 0.07 ^a,B^
	2	1.22 ± 0.03 ^b,A^	1.04 ± 0.01 ^b,B^
	3	0.47 ± 0.03 ^c,A^	0.34 ± 0.03 ^c,B^
	Total	4.31	3.52
Globulin	1	1.10 ± 0.09 ^a,B^	1.29 ± 0.02 ^a,A^
	2	0.52 ± 0.06 ^b,A^	0.60 ± 0.07 ^b,A^
	3	0.28 ± 0.08 ^c,A^	0.27 ± 0.02 ^c,A^
	Total	1.90	2.16
Prolamin	1	1.33 ± 0.02 ^a,A^	0.97 ± 0.06 ^a,B^
	2	0.76 ± 0.14 ^b,A^	0.44 ± 0.01 ^b,B^
	3	0.27 ± 0.04 ^c,A^	0.20 ± 0.00 ^c,B^
	Total	2.36	1.61
Glutelin	1	1.40 ± 0.17 ^a,A^	1.20 ± 0.09 ^a,A^
	2	0.68 ± 0.10 ^b,A^	0.74 ± 0.09 ^b,A^
	3	0.34 ± 0.00 ^c,B^	0.39 ± 0.02 ^c,A^
	Total	2.42	2.33
Total		10.99	9.62

^a–c^ Means in a column for a specific fraction with different superscripts differ significantly (*p* < 0.05). ^A,B^ Means in a row with different superscripts differ significantly (*p* < 0.05).

**Table 5:** Total flavonoid content (mg QE/g dry weight) in steam-distilled residue of coriander (SRC) and defatted steam-distilled residue of coriander seeds (DSRC) protein fractions.

Fractions	Extraction №	Content, mg QE/g Dry Weight	
		SRC	DSRC
Albumin	1	1.06 ± 0.02 ^a,A^	0.99 ± 0.04 ^a,A^
	2	0.79 ± 0.01 ^b,A^	0.62 ± 0.00 ^b,B^
	3	0.36 ± 0.01 ^c,A^	0.32 ± 0.00 ^c,B^
	Total	2.21	1.93
Globulin	1	0.61 ± 0.00 ^a,A^	0.59 ± 0.00 ^a,B^
	2	0.34 ± 0.00 ^b,A^	0.26 ± 0.00 ^b,B^
	3	0.17 ± 0.00 ^c,A^	0.16 ± 0.00 ^c,A^
	Total	1.42	1.01
Prolamin	1	0.32 ± 0.00 ^a,A^	0.30 ± 0.00 ^a,B^
	2	0.13 ± 0.00 ^b,A^	0.13 ± 0.00 ^b,A^
	3	0.06 ± 0.00 ^c,A^	0.05 ± 0.00 ^c,A^
	Total	0.51	0.48
Glutelin	1	0.62 ± 0.00 ^a,A^	0.66 ± 0.01 ^a,A^
	2	0.48 ± 0.01 ^b,A^	0.40 ± 0.01 ^b,B^
	3	0.25 ± 0.00 ^c,A^	0.20 ± 0.00 ^c,B^
	Total	1.35	1.26
Total		5.49	4.68

^a–c^ Means in a column for a specific fraction with different superscripts differ significantly (*p* < 0.05). ^A,B^ Means in a row with different superscripts differ significantly (*p* < 0.05).

**Table 6:** Scavenging effect of SRC and DSRC protein fractions on 2,2-diphenyl-1-picryl hydrazyl (DPPH) radical.

Fractions	Extraction №	Antiradical Activity, %	
		SRC	DSRC
Albumin	1	56.06 ± 0.27 ^a,B^	58.86 ± 0.25 ^a,A^
	2	53.16 ± 0.33 ^b,B^	56.43 ± 0.74 ^b,A^
	3	48.82 ± 0.34 ^c,B^	56.47 ± 0.50 ^b,A^
Globulin	1	46.11 ± 0.35 ^a,A^	25.32 ± 0.86 ^a,B^
	2	15.15 ± 0.23 ^b,B^	18.87 ± 0.64 ^b,A^
	3	12.36 ± 0.35 ^c,B^	15.67 ± 0.51 ^c,A^
Prolamin	1	25.48 ± 0.27 ^a,B^	32.01 ± 0.91 ^a,A^
	2	18.85 ± 0.88 ^b,B^	24.81 ± 0.33 ^b,A^
	3	15.49 ± 0.08 ^c,B^	21.69 ± 0.33 ^c,A^
Glutelin	1	14.50 ± 0.23 ^a,A^	10.46 ± 0.00 ^a,B^
	2	13.60 ± 0.28 ^b,A^	5.97 ± 0.95 ^b,B^
	3	10.88 ± 0.34 ^c,A^	1.43 ± 0.07 ^c,B^

^a–c^ Means in a column for a specific fraction with different superscripts differ significantly (*p* < 0.05). ^A,B^ Means in a row with different superscripts differ significantly (*p* < 0.05)

**Table 7:** Ferric Reducing Antioxidant Power (FRAP) of SRC and DSRC protein fractions.

Fractions	Extraction №	FRAP, µmol Fe^2+^/g Dry Weight	
		SRC	DSRC
Albumin	1	21.66 ± 0.37 ^a,A^	21.04 ± 0.72 ^a,A^
	2	9.95 ± 0.12 ^b,B^	11.02 ± 0.41 ^b,A^
	3	4.41 ± 0.11 ^c,A^	4.42 ± 0.06 ^c,A^
Globulin	1	7.39 ± 0.00 ^a,B^	8.28 ± 0.30 ^a,A^
	2	3.45 ± 0.13 ^b,A^	3.29 ± 0.06 ^b,A^
	3	1.63 ± 0.01 ^c,A^	1.59 ±0.05 ^c,A^
Prolamin	1	7.69 ± 0.01 ^a,B^	8.00 ± 0.11 ^a,A^
	2	3.32 ± 0.14 ^b,B^	3.72 ± 0.02 ^b,A^
	3	1.41 ± 0.01 ^c,B^	1.61 ± 0.06 ^c,A^
Glutelin	1	8.60 ± 0.05 ^a,B^	8.80 ± 0.06 ^a,A^
	2	5.79 ± 0.34 ^b,B^	6.55 ± 0.10 ^b,A^
	3	3.16 ± 0.03 ^c,B^	4.10 ± 0.06 ^c,A^

^a–c^ Means in a column for a specific fraction with different superscripts differ significantly (*p* < 0.05). ^A,B^ Means in a row with different superscripts differ significantly (*p* < 0.05)

**Table 8:** Hydroxyl radical scavenging activity of SRC and DSRC protein fractions.

Fractions	Extraction №	Inhibition, %	
		SRC	DSRC
Albumin	1	88.66 ± 0.80 ^a,A^	70.12 ± 0.00 ^a,B^
	2	81.23 ± 0.50 ^b,A^	64.40 ± 1.95 ^ab,B^
	3	71.81 ± 1.20 ^c,A^	62.82 ± 2.88 ^b,B^
Globulin	1	22.57 ± 3.07 ^a,B^	35.22 ± 2.31 ^a,A^
	2	18.13 ± 0.14 ^ab,B^	26.21 ± 0.40 ^b,A^
	3	14.48 ± 1.12 ^b,B^	23.65 ± 1.00 ^b,A^
Prolamin	1	NE	NE
	2	NE	NE
	3	NE	NE
Glutelin	1	NE	NE
	2	NE	NE
	3	NE	NE

^a–c^ Means in a column for a specific fraction with different superscripts differ significantly (*p* < 0.05). ^A,B^ Means in a row with different superscripts differ significantly (*p* < 0.05). NE: Not evaluated

**Table 9:** ABTS antiradical activity of SRC and DSRC protein fractions.

Fractions	Extraction №	ABTS Antiradical Activity, mM TE/g Dry Weight	
		SRC	DSRC
Albumin	1	17.79 ± 0.12 ^a,B^	18.69 ± 0.17 ^a,A^
	2	6.04 ± 0.12 ^b,B^	7.91 ± 0.11 ^b,A^
	3	2.37 ± 0.02 ^c,B^	2.71 ± 0.04 ^c,A^
Globulin	1	2.82 ± 0.15 ^a,B^	3.23 ± 0.08 ^a,A^
	2	0.99 ± 0.04 ^b,B^	1.20 ± 0.03 ^b,A^
	3	0.47 ± 0.04 ^c,A^	0.52 ± 0.01 ^c,A^
Prolamin	1	2.70 ± 0.07 ^a,B^	3.16 ± 0.06 ^a,A^
	2	0.95 ± 0.03 ^b,B^	1.34 ± 0.03 ^b,A^
	3	0.43 ± 0.01 ^c,B^	0.59 ± 0.01 ^c,A^
Glutelin	1	NE	NE
	2	NE	NE
	3	NE	NE

^a–c^ Means in a column for a specific fraction with different superscripts differ significantly (*p* < 0.05). ^A,B^ Means in a row with different superscripts differ significantly (*p* < 0.05).

**Table 10:** Cupric reducing antioxidant capacity (CUPRAC) of SRC and DSRC protein fractions.

Fractions	Extraction №	CUPRAC, mM TE/g Dry Weight	
		SRC	DSRC
Albumin	1	27.23 ± 0.40 ^a,B^	30.80 ± 0.36 ^a,A^
	2	12.43 ± 0.25 ^b,B^	13.40 ± 0.22 ^b,A^
	3	4.71 ± 0.09 ^c,B^	5.61 ± 0.10 ^c,A^
Globulin	1	6.00 ± 0.12 ^a,B^	7.71 ± 0.07 ^a,A^
	2	2.82 ± 0.03 ^b,B^	3.35 ± 0.03 ^b,A^
	3	1.32 ± 0.02 ^c,B^	1.42 ± 0.02 ^c,A^
Prolamin	1	8.86 ± 0.07 ^a,A^	6.94 ± 0.10 ^a,B^
	2	3.40 ± 0.05 ^b,A^	3.05 ± 0.08 ^b,B^
	3	1.63 ± 0.02 ^c,A^	1.39 ± 0.02 ^c,B^
Glutelin	1	11.81 ± 0.28 ^a,B^	12.89 ± 0.41 ^a,A^
	2	7.15 ± 0.11 ^b,B^	7.98 ± 0.18 ^b,A^
	3	4.42 ± 0.04 ^c,A^	4.01 ± 0.07 ^c,B^

^a–c^ Means in a column for a specific fraction with different superscripts differ significantly (*p* < 0.05). ^A,B^ Means in a row with different superscripts differ significantly (*p* < 0.05).

## Data Availability

The original contributions presented in this study are included in the article/Supplementary Materials. Further inquiries can be directed to the corresponding author.

## Supplementary Materials

The following supporting information can be downloaded at: https://www.mdpi.com/article/10.3390/foods15213993/s1, Supplementary Figure S1. Chromatograms of FAME standards and coriander fatty acids profile (enlarged); Supplementary Figure S2. Chromatograms of FAME standards and coriander fatty acids profile; Supplementary Figure S3. Chromatograms of methyl petroselenate and methyl oleate; Supplementary Figure S4. Chromatograms of methyl petroselenate and methyl oleate and coriander fatty acids profile.
