# Supplementary material for: Osborne Fractionation of Steam-Distilled Residue of Coriander (Coriandrum sativum L.) Seeds: Going Beyond Proteins
Source: Foods. 2026 Nov 7;15(21):3993. doi: 10.3390/foods15213993 (PMC13763559; doi:10.3390/foods15213993)
Supplement: Supplementary file 1 [file foods-15-03993-s001.zip › foods-4603040-supplementary.pdf]

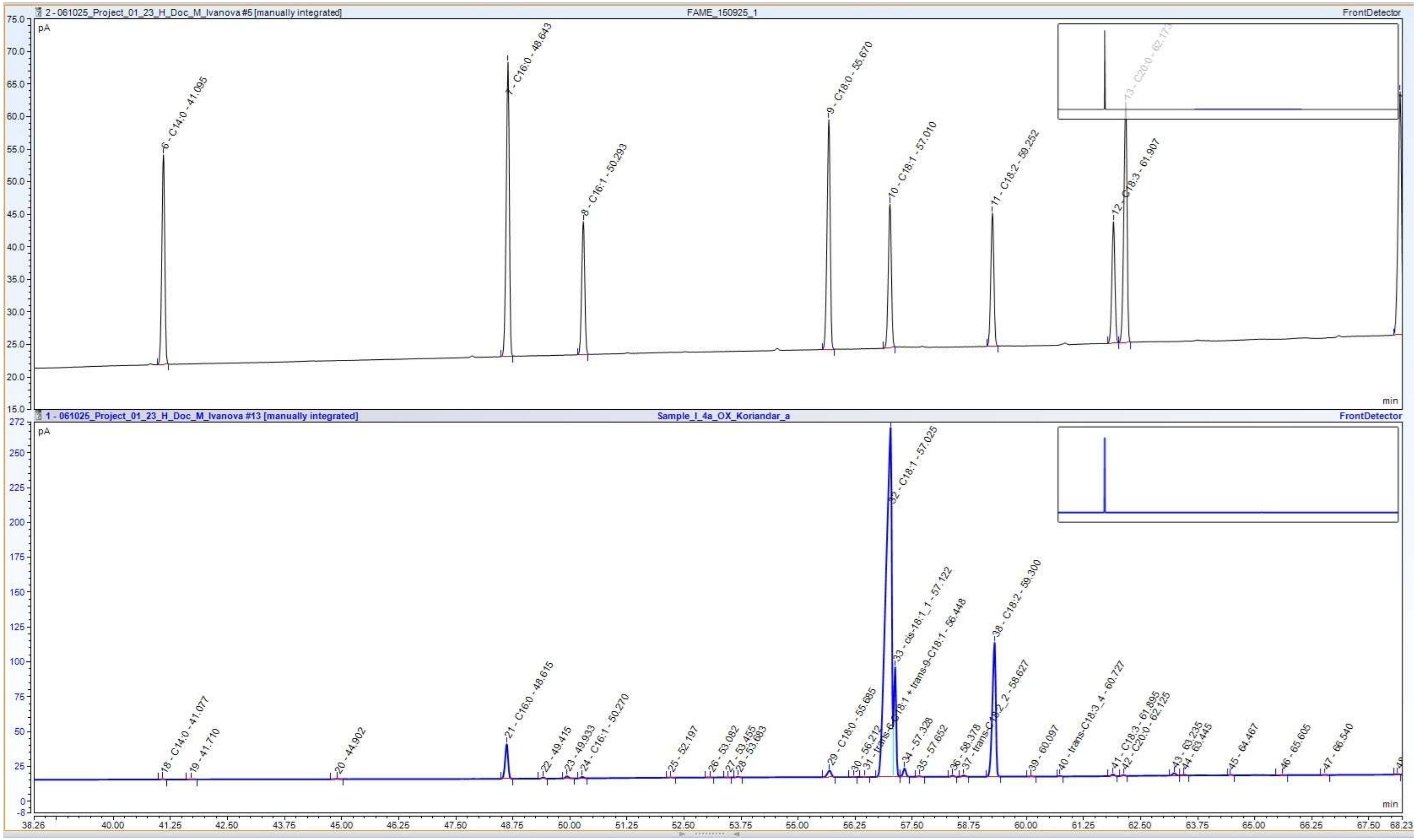

Supplementary Figure S1. Chromatograms of FAME standards and coriander fatty acids profile (enlarged)

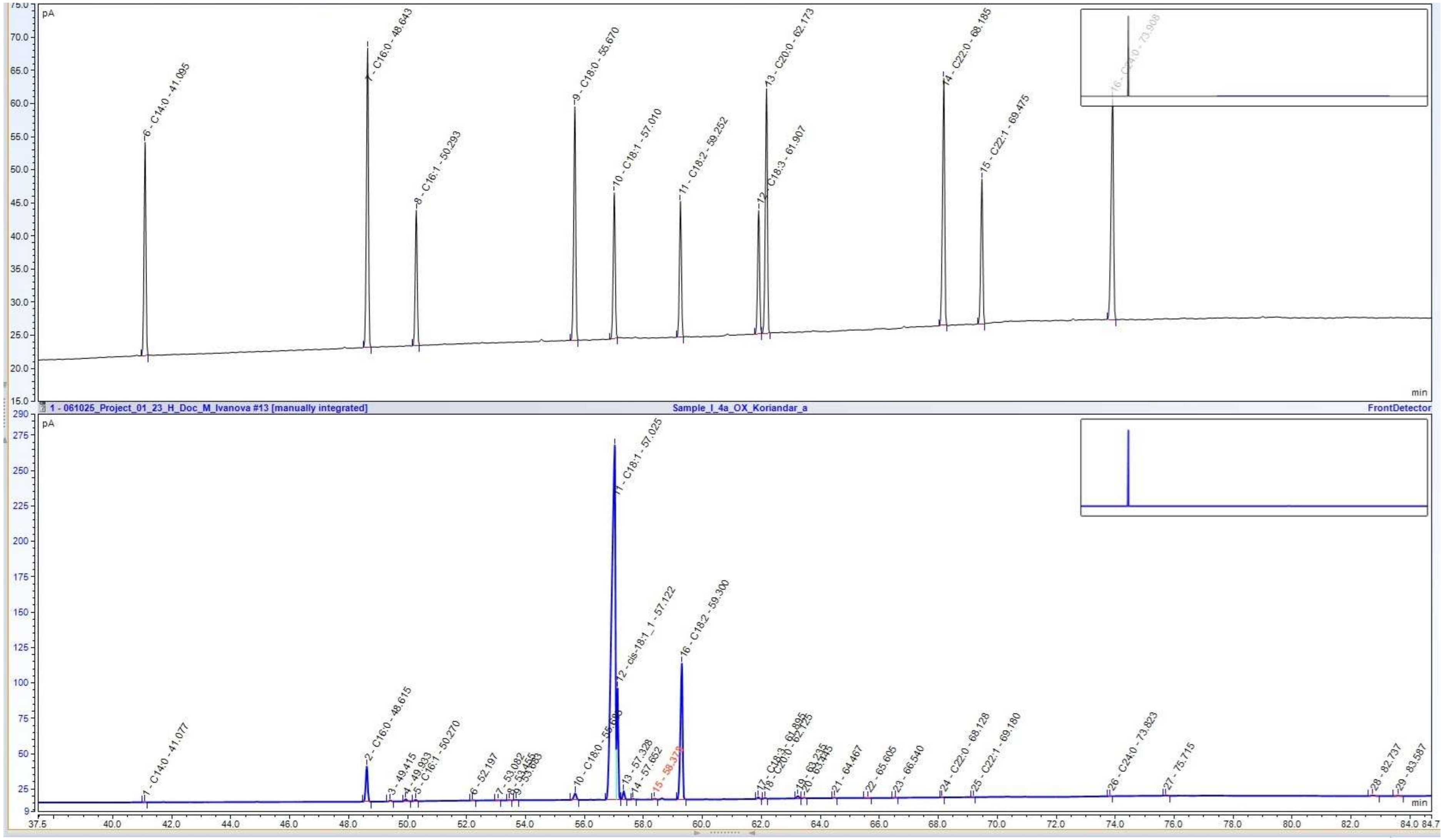

Supplementary Figure S2. Chromatograms of FAME standards and coriander fatty acids profile

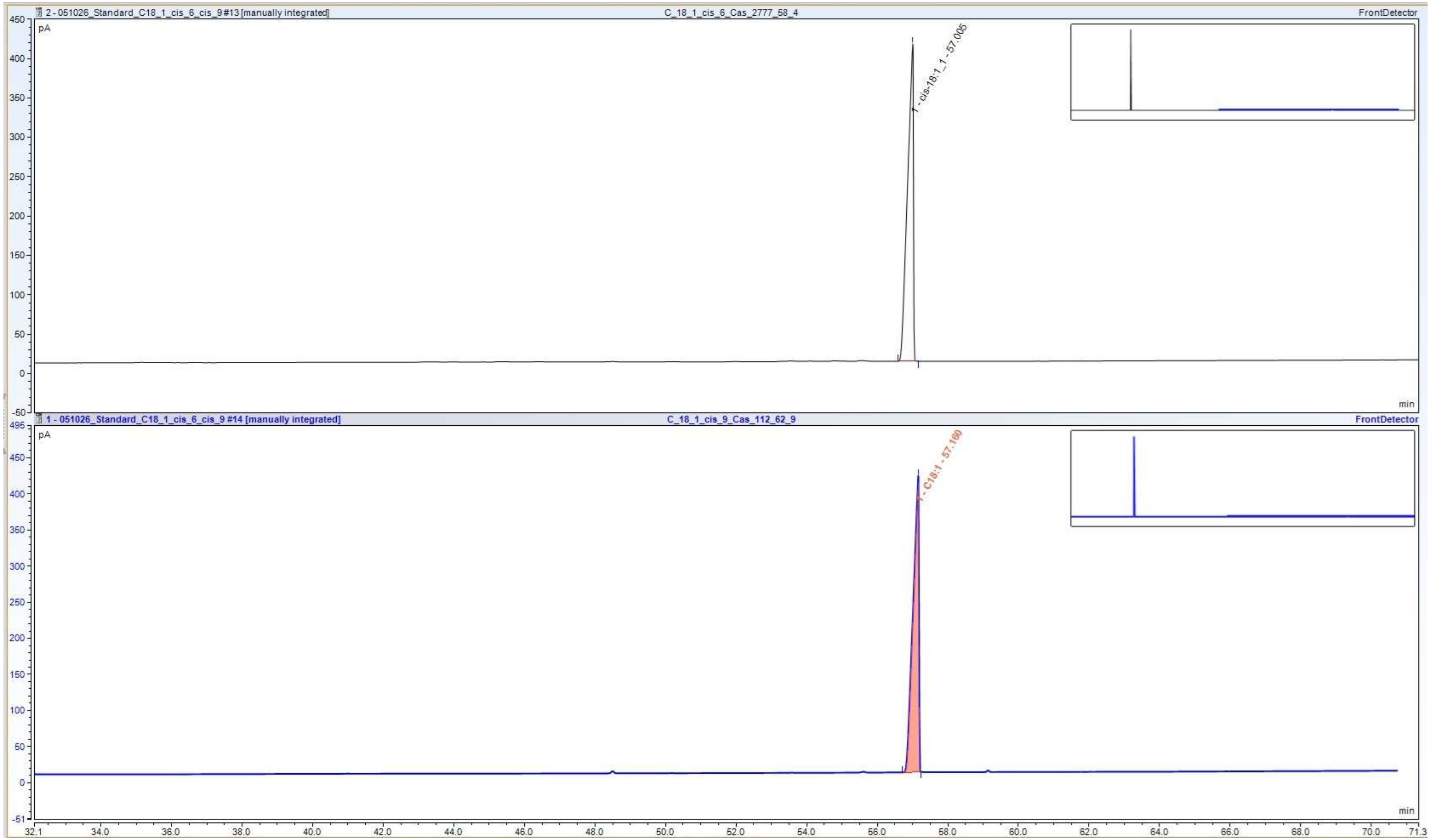

Supplementary Figure S3. Chromatograms of methyl petroselenate and methyl oleate

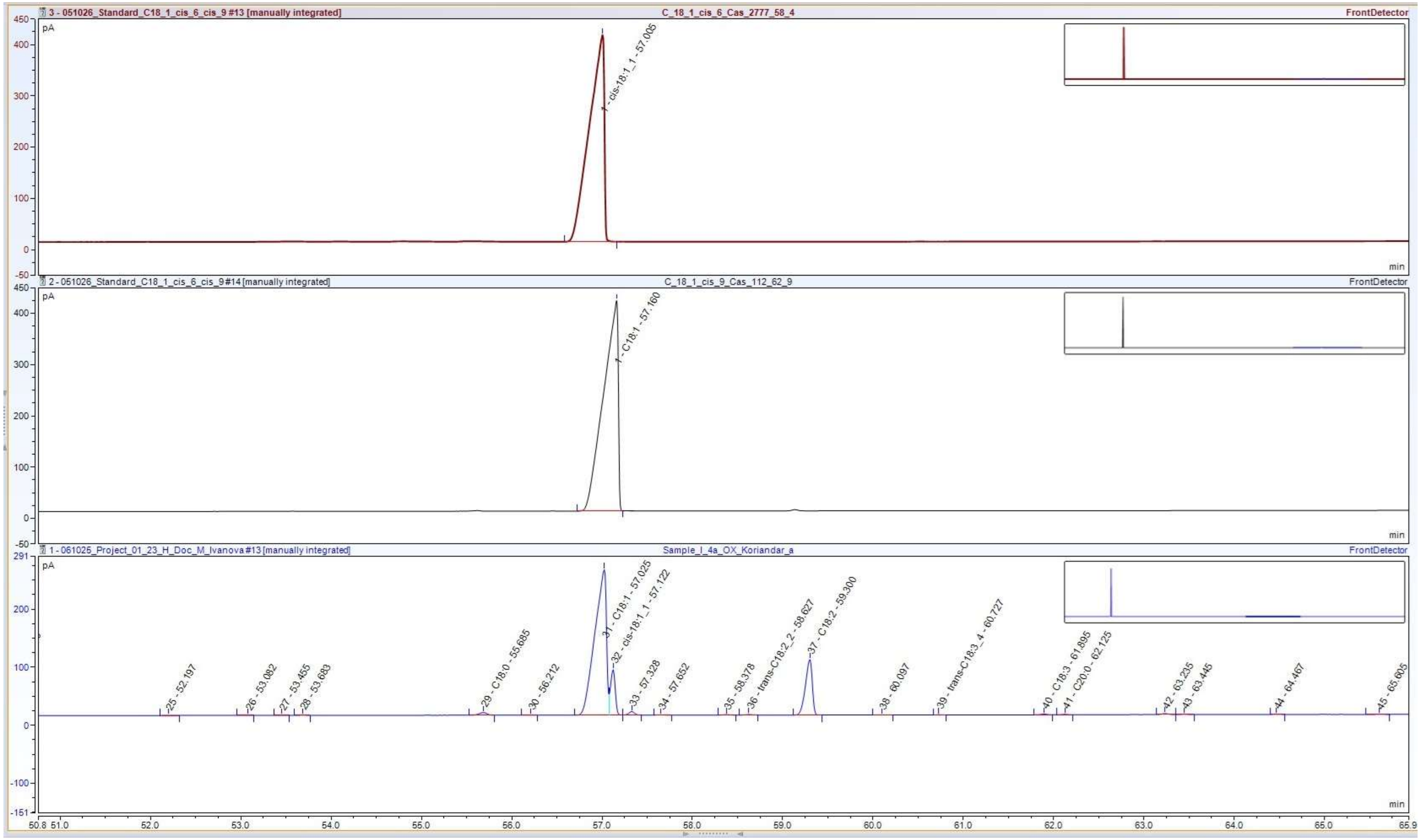

Supplementary Figure S4. Chromatograms of methyl petroselenate and methyl oleate and coriander fatty acids profile
